# An intronic SNP in the thyroid hormone receptor β gene is associated with pituitary cell-specific over-expression of a mutant thyroid hormone receptor β2 (R338W) in the index case of pituitary-selective resistance to thyroid hormone

**DOI:** 10.1186/1479-5876-9-144

**Published:** 2011-08-26

**Authors:** Anna Teresa Alberobello, Valentina Congedo, Hong Liu, Craig Cochran, Monica C Skarulis, Douglas Forrest, Francesco S Celi

**Affiliations:** 1 Diabetes, Endocrinology, and Obesity Branch, NIDDK-NIH Bethesda, Maryland, USA; 2 Department of Medicine and Sciences of Aging, University G D'Annunzio Chieti Italy; 3 Clinical Endocrinology Branch, NIDDK-NIH Bethesda, Maryland, USA

## Abstract

**Background:**

The syndrome of resistance to thyroid hormone (RTH) is caused by mutations in the thyroid hormone receptor β gene (*THRB*). The syndrome varies from asymptomatic to diffuse hypothyroidism, to pituitary-selective resistance with predominance of hyperthyroid signs and symptoms. The wide spectrum of clinical presentation is not completely attributable to specific *THRB *mutations. The *THRB *gene encodes two main isoforms, TR β1 which is widely distributed, and TR β2, whose expression is limited to the cochlea, retina, hypothalamus, and pituitary. Recent data demonstrated that in mice an intron enhancer region plays a critical role in the pituitary expression of the β2 isoform of the receptor. We thus hypothesized that polymorphisms in the human homologous region could modulate the pituitary expression of the mutated gene contributing to the clinical presentation of RTH.

**Methods:**

Screening and *in vitro *characterization of polymorphisms of the intron enhancer region of the *THRB *gene in the index case of pituitary-selective RTH.

**Results:**

The index case of pituitary-selective resistance is characterized by the missense R338W exon 9 mutation in *cis *with two common SNPs, rs2596623T and rs2596622C, located in the intron enhancer region of the *THRB *gene. Reporter gene assay experiments in GH3 pituitary-derived cells indicate that rs2596623T generates an increased pituitary cell-specific activity of the TR β2 promoter suggesting that rs2596623T leads to pituitary over-expression of the mutant allele.

**Conclusions:**

The combined coding mutation and non-coding SNP therefore generate a tissue-specific dominant-negative condition recapitulating the patient's peculiar phenotype. This case illustrates the role of regulatory regions in modifying the clinical presentation of genetic diseases.

## Background

Mutations in the *THRB *gene, encoding thyroid hormone receptor β (TRβ), result in the syndrome of resistance to thyroid hormone (RTH)[1,2]. RTH is a rare autosomal dominant disease with two main clinical presentations. The generalized RTH (GRTH) is characterized by elevated levels of TH, inappropriately normal or elevated levels of TSH [3], and a variable clinical presentation, ranging from asymptomatic to severe hypothyroidism [4,5]. A rarer form of RTH is characterized by selective resistance mostly limited to the pituitary (PRTH), with a prevalence of hyperthyroid symptoms since peripheral tissues are relatively less resistant to TH action but are exposed to elevated TH levels [6]. Most RTH mutations are localized in "hot spots" in the C-terminal coding exons of *THRB*, that generate dominant negative proteins with impaired ligand binding [7]. Despite striking differences among the clinical presentations, there is a lack of strict genotype-phenotype correlation and identical *THRB *gene mutations have been observed in PRTH or GRTH patients [8].

**Figure 1:**
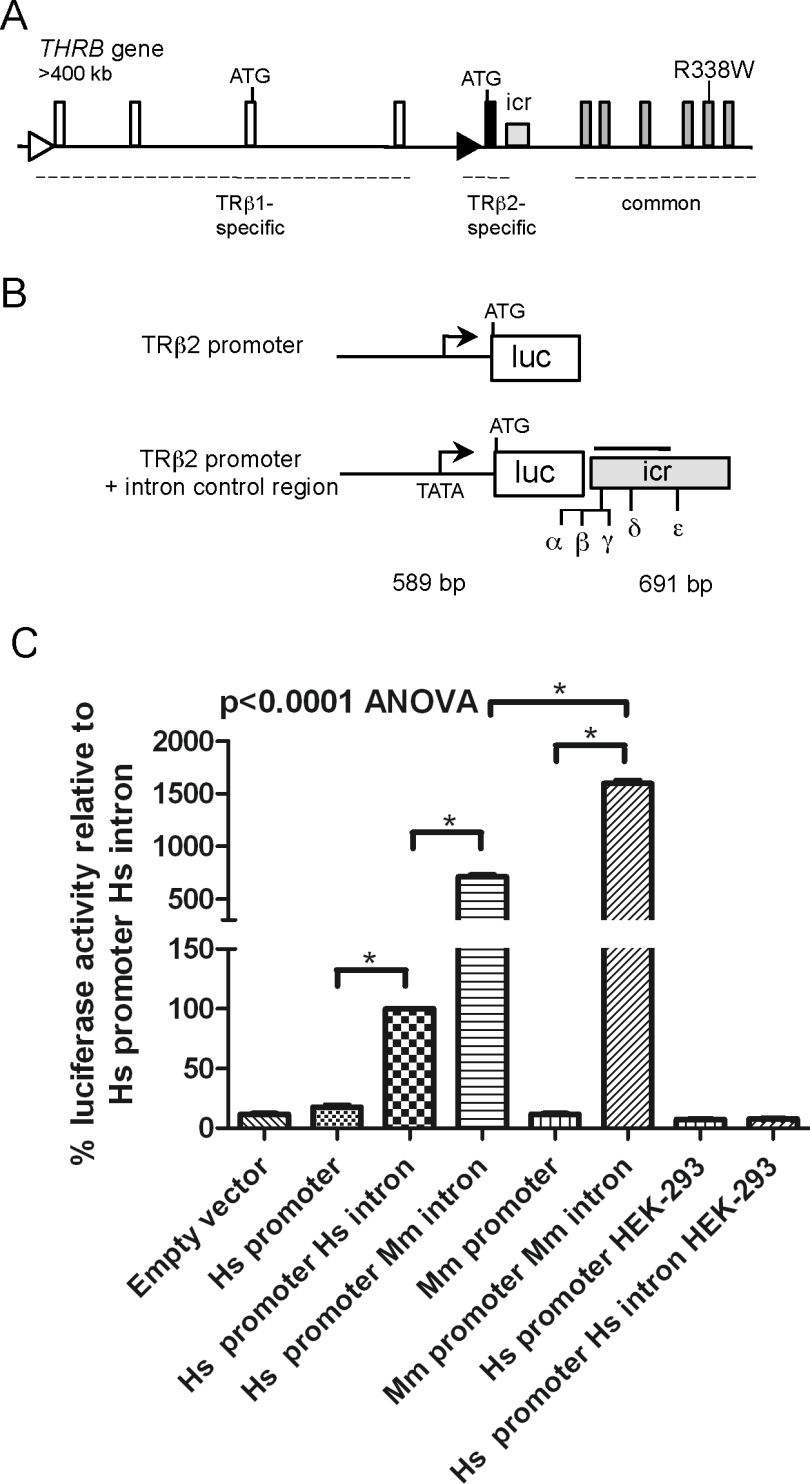
***THRB *gene structure, location and transcriptional activity of ICR SNPs**. **A**. Diagram of the *THRB *gene showing the origins of the TR β1- and TR β2-specific transcripts. The gray square represents the conserved 600 bp TR β2-specific intron control region (ICR), homologous to the mouse sequence. The location of the exonic R338W mutation in the index case of PRTH is marked by a vertical line. Major TR β1-specific 5' exons are shown, but not all variable untranslated sequences of TR β1 transcripts are included [24]. **B**, Luciferase reporter gene constructs, showing in the lower half of the panel the location of SNPs found in the ICR (rs6798561 α, rs17194828 β, rs2596623 γ, rs2596622 δ, rs77624520 ε). **C**, Transcriptional activity of the reporter constructs; genomic sequence origin, human (Hs, *Homo sapiens*), mouse (Mm, *Mus musculus*). Compared to the human promoter alone, the human promoter plus ICR gave a 10-fold increase in pituitary cell-specific luciferase expression. Constructs containing the murine promoter and ICR are included as positive controls for ICR activity. Analysis of the chimeric reporter (human promoter + mouse ICR) indicated that the ICR activity is conserved. No activity of the ICR was observed in kidney-derived HEK-293T cells (*see text for details*). * = significant on Tukey's post-hoc analysis.

The *THRB *gene expresses N-terminal variant TR β1 and TR β2 isoforms from specific promoters (Figure 1A). TR β1 is relatively widely expressed whereas TR β2 is restricted with main sites of expression in the anterior pituitary, hypothalamus, cochlea and retina [9,10]. A conserved 600 bp intron control region (ICR) directs expression of TR β2 in the pituitary and retina in transgenic mice [10]. In this study we investigate SNPs in the ICR of the human *THRB *gene in RTH. The screening of the ICR in our RTH cohort demonstrated the presence of a novel and four common SNPs. We also report that in the index case of PRTH a common SNP in the ICR stimulates over-activation of the TR β2 promoter in pituitary cells *in vitro*. Moreover, we determined that this SNP is linked in *cis *with the known R338W coding mutation. We propose that this genetic "double hit" of coding and non-coding changes in the same *THRB *allele, ultimately generates a tissue-specific dominant negative condition underlying this case of PRTH.

## Methods

### Patients and specimens

All patients provided an informed consent to the participation in the study and the collection of genetic material. Medical records of 45 RTH patients followed at the NIH Clinical Center since 1976, for whom DNA samples were available, were reviewed [8]. The cases were categorized as either GRTH or PRTH; the diagnosis of PRTH was made if at least two of the following findings were described in the medical records: resting tachycardia, resting energy expenditure >110% of predicted by the Harris-Benedict formula [11], hyperactive behavior, and low body mass index (<20 kg/m^2 ^in adults or <5^th ^percentile in children).

### Genomic DNA amplification and sequencing

Exons 9, 10, and a 607 bp ICR fragment of the *THRB *gene of the index case of pituitary selective RTH were amplified and screened for mutations by direct sequencing. Each PCR was performed using Platinum^® ^Taq from Invitrogen (Carlsbad, CA) using the following primers: exon 9: 5'-GAAAACCATGGGCTCAAAGA-3', and 5'-AGCGCTAGACAAGCAAAAGC-3'; exon 10: 5'-TAAAGGCCTGGAATTGGACA-3' and 5'-TCCCTCCCAACACAAAGAAA-3'; ICR: 5-TGAGGTACATTGAACATGTGC-3' and 5'-ACTGAACACCTGTTTATGGTC-3'. The sequences were analyzed and visually inspected using Sequence Scanner 1.0 (Applied Biosystems, Foster City, CA).

### Reporter gene constructs

A 589 bp human TR β2 promoter fragment homologous to the mouse promoter [12] and a 691 bp ICR fragment were directionally cloned into a luciferase reporter construct in a pRep4 episomal vector [10]. Fragments were amplified by PCR from a healthy, unrelated subject using primers as follows: promoter: 5'-GGT-Nhel-TCATTACTACTGGATTTTC-3' and 5'-TGG-HindIII-GTTTCCCTGGTTCAGTTTC-3'; ICR: 5'-GTT-BamHI-TAATGGAAACATTTGAGGT-3'and 5'-GAA-SalI-AGATGAAGCAATTATGAAC-3'. This construct was used as a template to generate mutations in the ICR using QuikChange™ Site-Directed Mutagenesis (Stratagene, La Jolla, CA) (Figure 1B). A promoterless construct containing the ICR, and a construct lacking the ICR but containing the TR β2 promoter were generated as negative controls. A chimeric construct contained the human promoter and a mouse ICR (730bp ICR fragment of mouse *Thrb *gene obtained by PCR) (NC_00085.5) as described [10]. All constructs were sequenced in entirety.

### Transfection and luciferase assay

Media, sera, antibiotics for cell culture, and transfection reagents were purchased from Invitrogen (Carlsbad, CA). GH3 or HEK-293T cells (6 × 10^3 ^cells/cm^2^) were grown in DMEM supplemented with 10% (v/v) FBS and 2 mM glutamine. Cultures were maintained at 37°C in a humidified atmosphere containing 5% (v/v) CO_2_.

Transfections with 200 ng of each luciferase reporter plasmid, 20 ng of pSV-RL Renilla internal control plasmid, and empty pcDNA-3 carrier DNA were performed with Lipofectamine™. Thirty-six hours after transfection, the cells were kept in serum-free medium overnight before harvesting. Luciferase activity was assayed with a dual-luciferase system and normalized to renilla activity (Promega, Madison, WI).

### Statistical Analysis

The frequency of categorical variables (clinical characteristics and generalized vs. pituitary-selective RTH) was analyzed by chi-square, while continuous variables (luciferase reporter assay data) were assessed by one-way ANOVA with Tukey's post-hoc analysis. An α error of 0.05 was considered the threshold for statistical significance.

## Results

### Case presentation

This case was originally described by Gershengorn and Weintraub in 1975 [6]. This female patient, currently 53-years old, was at age 7 referred to the NIH Clinical Center because of goiter and hyperthyroidism. The evaluation showed increased thyroid hormone and TSH levels, with a net prevalence of hyperthyroid signs, including tachycardia, increased basal metabolic rate, and brisk reflexes. Further studies demonstrated the absence of a pituitary adenoma and the rise in TSH following the injection of TRH. The clinical picture was interpreted as "Thyrotropin-induced hyperthyroidism caused by a selective pituitary resistance to thyroid hormone" [6]. The patient was initially treated with methimazole, but the therapy was discontinued because of urticaria, and a sub-total thyroidectomy was performed. The symptoms recurred after surgery and she was treated with propylthiouracil and, at age 32, with radioactive iodine ablation. Since then, the patient has been maintained on levothyroxine and beta-blockers with the therapeutic goal of clinical euthyroidism and a TSH level in the high-normal range. The patient is doing well and has a 20-year old non-affected daughter in good health. Her therapy consists of levothyroxine 175 mcg daily (3.8 mcg/Kg), Atenolol 25 mg daily; her most recent laboratory data are: fT4 3.8 ng/dL (0.8-1.5), TSH 5.05 mcIU/mL (0.4-4.0), cholesterol 199 mg/dL (<200), triglycerides 81 mg/dL (<150), HDL 60 mg dL (>60), LDL 123 mg/dL (<100), SHBG 60 nmol/l (18-114), ACE 41 U/L (16-52). A DEXA scan showed a T-score of -0.6.

### Assignment of ICR haplotype in the index case of PRTH

**Figure 2:**
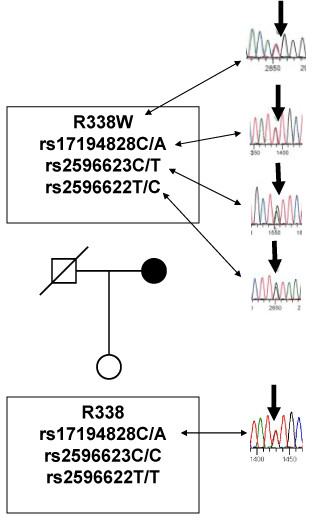
**Haplotype assignment of the index case of PRTH**. Direct sequencing the ICR and exon 9 of the THRB demonstrated that the patient carried the heterozygous genotypes C/A in rs17194828, C/T in rs2596623, and T/C in rs2596622, and the R338W mutation. Conversely, the unaffected daughter carries the heterozygous genotype C/A in rs17194828. The data thus indicate that the patient's mutant W338 allele is in *cis *with the rs2596623 T and rs2596622 C allele and the unaffected daughter inherited the maternal haplotype rs2596623C-rs2596622T, and R338.

The index case of PRTH, affected by a previously characterized mutation in the exon 9 (R338W)[13], carried the heterozygous genotypes C/A in rs17194828, C/T in rs2596623, and T/C in rs2596622. In order to deduce the patient's haplotype, the ICR and the TR β exon 9 of her unaffected daughter were genotyped. Direct sequencing showed that the daughter is homozygous for the wild type R338 allele, heterozygous for the rs17194828 SNP (C/A genotype), and homozygous for the rs2596623 and rs2596622 SNPs (C/C and T/T genotypes, respectively). The data thus indicate that the patient's mutant W338 allele is in *cis *with the rs2596623T allele and rs2596622 C allele, and the unaffected daughter inherited the maternal haplotype rs2596623C-rs2596622T, and R338 (Figure 2). Due to the unavailability of paternal DNA, we cannot assign the genotype for the rs17194828 SNP. Direct sequencing of a cohort of 45 genomic samples of RTH NIH cohort patients (Table 1) [8] revealed the presence of two additional SNPs, the previously described rs6798561G/A, and a novel one, rs77624520G/C present in a single case. A schematic representation of the ICR SNPs distribution in the *THRB *gene is reported in figure 1. Nο statistical difference was found between GRTH and PRTH in the prevalence of each of the SNPs (Table 2).

**Table 1:** Clinical presentation and mutations in the exons 9 and 10 of the *THRB *gene in the NIH RTH cohort

*Exon*	*Amino acid change*	*Form*	*n. subjects*
9	A317T	GRTH	3

9	R320L	GRTH	1

9	D322H	GRTH	3

9	G332R	GRTH	1

9	R338W	GRTH	2
		
		PRTH	2

9	G345S	GRTH	3
		
9	G347A	GRTH	3

		PRTH	1
		
10	R383H	GRTH	1

10	R438H	GRTH	1

10	M442V	GRTH	1
		
		PRTH	1

10	M442R	GRTH	2
		
		PRTH	1

10	FRSH 448	PRTH	1

10	P453H	GRTH	7
		
		PRTH	2

Not found	n/a	GRTH	6
		
		PRTH	3

**Table 2:** Prevalence of the SNPs in the intron control region of the *THRB *gene in the NIH RTH cohort

SNP ID	Generalized RTH		Pituitary RTH		Odds ratio [CI]	p value
	Subjects	Sex	Subjects	Sex		
	n = 34	16 M	n = 11	8 M		
		18 F		3 F		
**rs6798561**	n	%	n	%		
Genotype						
GG	33	97.0	11	100		
GA	1	3	0	0	n.a	n.a
AA	0	0	0	0		

**rs17194828**	n	%	n	%	CC *vs*. CA plus AA	
Genotype						
CC	28	82.3	7	63.6		
CA	6	17.6	4	36.4	2.7 [0.6 to 12.1]	0.228
AA	0	0	0	0		

**rs2596623**	n	%	n	%	CC *vs*. CT plus TT	
Genotype						
CC	13	38.2	3	27.3		
CT	18	52.9	7	63.6	1.7 [0.4 to 7.4]	0.720
TT	3	8.82	1	9.1		

**rs2596622**	n	%	n	%	TT *vs*. TC plus CC	
Genotype						
TT	15	44.1	3	27.3		
TC	16	47.0	8	72.7	2.1 [0.5 to 9.3]	0.482
CC	3	8.8	0	0		

**rs77624520**	n	%	n	%		
Genotype						
GG	33	97.0	11	100		
GC	1	3	0	0	n.a	n.a
CC	0	0	0	0		

The SNPs are listed according to the location on the chromosome as reported on Ensembl database.

### Transcriptional enhancer function of the human ICR

Analyses of a luciferase reporter construct containing the human TR β2 promoter demonstrated that in pituitary-derived GH3 cells, the human ICR stimulated a 10-fold increase in luciferase expression compared to a reporter carrying the promoter alone (p < 0.0001). In a chimeric reporter, the murine ICR also stimulated the human promoter indicating that ICR function was conserved between species, consistent with the high sequence conservation. Compared to the human ICR, the murine ICR stimulated a greater degree of enhancement of luciferase activity (p < 0.0001) under the assay conditions used (Figure 1C). Similarly to previous observations with murine sequences, no enhancer activity was observed in kidney-derived HEK-293T cells, demonstrating that the activity of the human ICR is cell type-specific.

### Enhancer function of ICR with different SNPs

**Figure 3:**
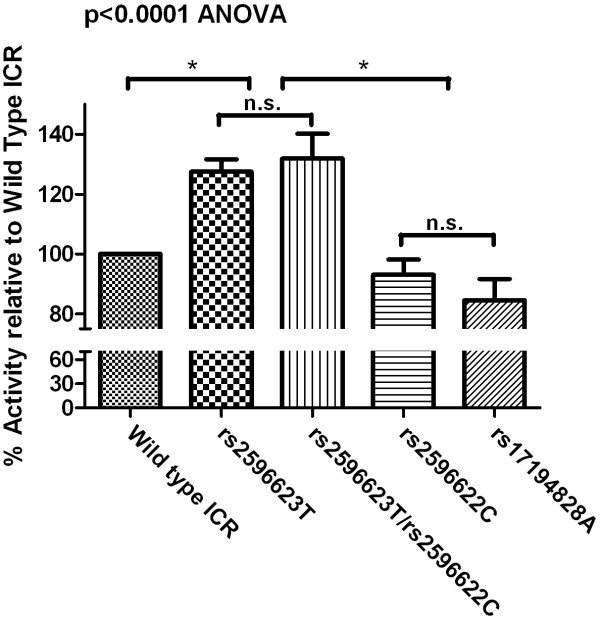
**Modulatory effects of SNPs on the expression of the TR β2 promoter-luciferase-ICR reporter construct**. The SNPs detected in the index case of PRTH were introduced in the reporter gene construct by site-directed mutagenesis. As compared to the "wild type" sequence, rs2596623T gave a significant 30% increase in transcriptional activity. A similar increase in transcriptional activity was observed when the cells were transfected with the rs2596623T/rs2596622C naturally occurring haplotype. Transfections of the rs2596622C or rs17194828A SNPs alone did not result in a significant change in the transcriptional activity of the reporter gene construct. No difference in transcriptional activity was observed after transfection with SNPs rs6798561A and rs77624520C (*data not shown*) (*see text for details*). The results were confirmed by three independent preparations of reporter plasmids that were tested at least thrice with triplicate points determined for each assay * = significant on Tukey's post-hoc analysis.

To evaluate the effects of ICR SNPs on TR β2 promoter activity, we generated luciferase reporter constructs containing the SNPs observed in the NIH RTH cohort. We also generated a rs2596623T-rs2596622C construct replicating the ICR haplotype of the PRTH index case. Compared to the ancestral allele the rs2596623T produced a significant 30% increase in luciferase expression (p < 0.0001), suggesting that the rs2596623T increased transcriptional activity of the TR β2 promoter. Compared to rs2596623T alone, the rs2596623T-rs2596622C haplotype resulted in a marginal, non-significant increase in transcriptional activity (p = 0.543), indicating that the increase in transcriptional activity is solely due to the rs2596623T (Figure 3). The other SNPs did not significantly change expression levels of luciferase.

## Discussion

Since the original description of RTH [1], and the subsequent description of the PRTH form [6], many investigators have attempted to demonstrate a correlation between genotype and phenotype. Although some mutations in the *THRB *gene may associate preferentially with PRTH, and among them R338W appears particularly frequent, patients sharing the same mutation can present the entire range of symptoms associated with the various forms of RTH [5,13]. Furthermore, although the clinical presentation tends to segregate within the kindred, there is no absolute concordance, and even among siblings the phenotype differences can be remarkable [14]. *In vitro *experiments demonstrated that the R338W mutant receptor has reduced affinity for TH and a dominant negative activity typical of RTH syndrome [13,15]. Some *in vitro *data suggest that R338W selectively impairs TR β2 rather than TR β1 isoform function [16], but this finding does not explain completely the clinical presentation since R338W is also associated with GRTH. Machado and co-workers reported that a naturally occurring mutation (R429Q) associated with PRTH produces a mutant TR β product that is specifically defective as a transcriptional repressor, while retaining relatively normal activator function [17]. This particular mutation is thus in keeping with a selective PRTH, since a main action of thyroid hormone in the thyrotroph is the inhibition of *TSH *β-subunit gene transcription. Taken together, these clinical and laboratory observations suggest that various factors, regardless of the particular *THRB *mutation, contribute to the clinical presentation of RTH.

Our hypothesis that ICR mutations play a role in the RTH presentation was further supported by the growing recognition that sequence variations in *cis*-acting control regions determine differences in gene expression levels. Furthermore, association studies have identified non-coding sequences as a risk factor for various diseases [18,19]. We have shown that the human ICR, like the murine ICR, stimulates TR β2 promoter activity in pituitary- but not in kidney-derived cell lines. The somewhat weaker activity of the human ICR than the murine ICR may reflect bias in the assay which was performed in rodent GH3 cells. Alternatively, there may be real species differences in the magnitude of the response since thyroid homeostasis parameters differ in degree between rodents and humans, as indicated for example by their different serum ratios of T3/T4 [20], or by different patterns of expression of deiodinase type-2 [21].

Our study of the NIH RTH cohort demonstrated four known SNPs and one novel SNP in the human ICR. The data indicate that in the index case of PRTH a specific ICR haplotype resides in *cis *to the R338W *THRB *gene pathogenic mutation. Further, when we tested the activity of each individual SNP, only rs2596623T gave a significant increase in reporter gene expression. rs2596623 resides near the 5' boundary of a highly conserved 380 bp sequence in the ICR that is sufficient to direct TR β2 promoter activation in the pituitary but not retina *in vivo *in transgenic mice [10]. We speculate that rs2596623T enhances the recruitment or binding stability of a transcription factor complex that activates the TR β2 promoter in the pituitary.

Our finding tend to support the hypothesis that rs2596623T stimulates pituitary over-expression of the pathogenic R338W TR β2 protein, tilting the balance of expression between the wild type and R338W mutant *THRB *alleles in a tissue-specific fashion. This dominant negative condition would result in a relatively increased resistance in the pituitary as compared to the peripheral tissues, recapitulating the patient's phenotype. Indeed, of the four unrelated RTH patients who carry the R338W mutation in the NIH RTH cohort (Table 1), the PRTH phenotype is limited to the two cases heterozygous for rs2596623 (C/T). For the second heterozygous case, due to lack of genetic material from relatives, we are unable to indicate whether rs2596623T resides in *cis *with the pathogenic R338W mutation. Nonetheless one could speculate that in the two R338W GRTH cases the homozygosity at rs2596623 (C/C or T/T) results in "balanced" expression of the wild type and mutant receptors in the pituitary, not dissimilar from peripheral tissues.

No statistical association was found between any SNP and the phenotypes. This is not surprising because of the relatively small number of subjects and the lack of genetic material that would allow us to determine haplotypes in most cases.

Gene expression levels are heritable and several human diseases have been associated with changes in enhancer sequences rather than coding exons. For example, a polymorphic intron sequence that increases *COL1A1 *transcription has been associated with changes in bone mineral density and increased incidence of osteoporotic bone fractures [22]. Another mutation that decreases activity of an intronic enhancer in the *RET *gene and has been associated with some cases of Hirschsprung disease [23]. The case of PRTH we describe is unique in suggesting a genetic "double hit" that combines an enhancer SNP and a coding exon change on a single *THRB *allele.

## Conclusions

The molecular characterization of the index case of PRTH is unique in illustrating a genetic "double hit" that combines an enhancer SNP and a coding exon mutation on a single *THRB *allele resulting in the modulation of the clinical presentation.

## Competing interests

The authors declare that they have no competing interests.

## Authors' contributions

ATA, carried out the *in vitro *studies, and wrote the manuscript draft. VC, contributed to the *in vitro *studies and analyzed the clinical data. HL, designed the reporter gene assay and contributed to the *in vitro *studies. CSC, MCS, contributed to the collection and analysis of the clinical data. DF contributed in the design of the study and in the interpretation of the data, and contributed to the writing of the manuscript. FSC, conceived the study, analyzed the data and supervised the writing of the manuscript.

All authors read and approved the final manuscript.
